# Development and preliminary validation of a Chinese Physical Activity Parenting Practices Scale (3–6 years)

**DOI:** 10.3389/fpsyg.2025.1560244

**Published:** 2025-04-17

**Authors:** Ren Na, Ying Liang, Haiyue Zhang, Zhe Yang, Nan Li, Wei Zhang, Han Tang, Weiliang Ye, Linyuan Zhang, Xun Jiang, Lei Shang

**Affiliations:** ^1^Department of Health Statistics, School of Public Health, Air Force Military Medical University, Xi'an, China; ^2^Department of Pharmacy and Pharmaceutical Management, Air Force Military Medical University, Xi'an, China; ^3^School of Nursing, Air Force Military Medical University, Xi'an, China; ^4^Department of Pediatrics, Tangdu Hospital, Air Force Military Medical University, Xi'an, China

**Keywords:** physical activity, validity, reliability, measurement, parents, children

## Abstract

**Background:**

This study aimed to develop a scale to assess the physical activity (PA)-related parenting practices of Chinese parents of children aged 3–6 years based on general parenting theory.

**Methods:**

A pool of scale items (123 items) was constructed based on a literature review and in-depth personal interviews. The pretest scale (60 items) was developed using Delphi correspondence and a presurvey. After two rounds of item screening of the pretest scale using exploratory factor analysis (EFA) and analysis of variance, we deleted 30 items. We ultimately developed a formal version of the Chinese Physical Activity Parenting Practices Scale (CPAPPS) using the remaining 30 items. We examined the structure of the scale using factor analysis and evaluated its reliability, validity, and discriminant ability using data from 899 parents of children aged 3–6 years.

**Results:**

The CPAPPS includes 30 items in 6 dimensions scored on a 5-point Likert scale. The 6 dimensions are education, autonomy promotion, modeling, demands, expectations, and rewards. Both exploratory and confirmatory factor analyses confirmed the construct validity of the scale. Furthermore, the scale had adequate internal consistency, split-half reliability, test–retest reliability, and concurrent validity. Parents younger than 30 scored significantly lower on the demand dimension than parents aged 40–50 (*p* < 0.05). The differences in rewards and expectations between parents of different ethnicities were statistically significant (*p* < 0.05). Compared with married parents, parents who were currently single had lower scores for education, rewards, modeling, and autonomy promotion (*p* < 0.05). There was a significant difference in scores across all dimensions between parents with different places of residence (*p* < 0.05).

**Conclusion:**

The CPAPPS satisfies the conditions for reliability and validity in accordance with psychometric requirements. The scale can be employed to evaluate the characteristics of Chinese parents’ physical activity-related parenting practices and to design family-based PA interventions.

## Background

Insufficient physical activity (PA) is now a global public health problem. The World Health Organization (WHO) reported that more than 80% of children and adolescents globally do not meet the WHO’s recommendation of completing at least 60 min of moderate- to vigorous-intensity PA daily (Bull et al., 2020). Although globally comparable data for younger children are not available (Willumsen and Bull, 2020), in China, a national report revealed that the rate of physical inactivity among children and adolescents was 84.3% (Zhang and Wang, 2012). It is well known that physical inactivity is a significant risk factor for many chronic diseases (Hansford et al., 2022). A survey on the nutritional status and chronic disease status of Chinese residents revealed that in 2020, the overweight and obesity rates for children and adolescents aged 6–17 years and under 6 years in China were 19 and 10.4%, respectively; the incidence of single behavioral abnormalities in children ranged from 10 to 20%; the prevalence of hyperactivity disorders, autism, learning disabilities, and behavioral disorders among children increased; and unhealthy lifestyles were prevalent (Yu and Zou, 2023). Chronic diseases such as hypertension, diabetes mellitus, dyslipidemia, and fatty liver, which typically appear only in adulthood, now appear in overweight and obese children and adolescents and indicate a trend toward chronic diseases at a younger age (Ding et al., 2016).

Parents are comparators and constructors of children and adolescents. Both multigenerational transmission theory and social-ecological systems theory suggest that, in contrast to other distal factors, parents exert a direct, enduring, and distinctive influence on the lifelong behaviors and attitudes of children and adolescents about PA (Eisenberg et al., 2014; Hu et al., 2021; Kennedy et al., 2021). In the context of parenting, parents influence children’s PA through multifaceted behavioral strategies. Researchers reported that parental encouragement continued to increase children’s PA 5 years later (Bauer et al., 2008). A systematic review also revealed that even when parent–child closeness is low, parents can still impact individual children’s behavior by supporting organized PA (Petersen et al., 2020). Logistical support, such as transporting children to and from PA and purchasing relevant equipment and gear, also facilitates the PA of children and adolescents (Laird et al., 2016).

Furthermore, regular parental exercise behaviors play a significant role in modeling PA in children and adolescents. Predictions of children’s participation in moderate to vigorous PA increase from 67 to 74% when parents increase the number of moderate to vigorous PA sessions per week from 0 to 3 (Isgor et al., 2013). Research reported that parental modeling led to a perceived increase in children’s sense of security and that the initial parental gift of a sense of participation influenced children’s enjoyment of PA (Xu and Gao, 2018). It has been suggested that parental control, restrictions, and supervision are associated with children’s PA, but in some cases, the results are contradictory (Trost et al., 2013; Hutchens and Lee, 2018). In a pre-conference session at the 2012 International Society for Behavioral Nutrition and Physical Activity (ISBNA) annual conference, experts from several countries defined physical activity parenting practices (PAPP) as the behavioral strategies used by parents to socialize their children’s PA (Mâsse et al., 2017). The early identification of problems with PAPP and the subsequent planning and development of parent-level interventions to create a supportive environment for children is vital for the development of PA habits in children. Developing a tool that can help identify problems with PAPP and objectively assess the severity of these problems is critical.

Since the 1990s, several instruments have been developed to assess PAPP. However, early studies were characterized by the inconsistent or non-existent adoption of theory in the construction of conceptual frameworks and a lack of agreed-upon measures, resulting in many incoherent and inconsistent measurement constructs between scales as well as measurements that are difficult to compare (Sleddens et al., 2012; Davison et al., 2013). In response to this issue, a pre-conference session at the 2012 annual meeting of the ISBNA recommended that follow-up research should integrate parenting dimensions from the general parenting literature into the conceptualization of PA parenting to promote the standardization of PAPP measures (Davison et al., 2013).

Three main approaches characterize the general parenting domain: (1) responsive, which refers to the extent to which parents help children develop individuality and self-assertion through the use of warmth, support for autonomy, and rational communication; (2) controlling or demanding, which refers to the extent to which parents exert an influence on children through directive, restrictive, and punitive parenting to force the child to satisfy parental demands; and (3) structural, which refers to parents’ organization of the child’s social and physical environment to promote the development of competence (Slater and Power, 1987; Conger, 2009; Power, 2013).

Based on this framework, Vaughn et al. (2013) developed the Physical Activity Parenting Practices Scales (PAPPS) (ages 2–5); O'Connor et al. (2014) compiled the Preschoolers’ Physical Activity Parenting Practices (PPAPP) (ages 3–6); Suen et al. (2017) developed the Physical Activity Parenting Practices for Preschoolers-Hong Kong questionnaire (PAPPP-HK); and in 2020, scholars from six countries—Australia, Canada, England, Scotland, the Netherlands, and the United States—developed the Physical Activity Parenting Practices item bank (PAPP-IB) (5–12 years) (Mâsse et al., 2020). Since parenting practices are deeply influenced by traditional culture and social conditions, these practices have inherited cultural characteristics. Therefore, the items in these scales may not be cross-culturally universal and may easily overlook phenomena and behavioral traits that are considered meaningful in Chinese culture. For example, although Hong Kong is a city in China, it is located on the coast and has a more developed economy. Many of the items in the PAPPP-HK, such as “How often do you take your child to the beach?” and “How often do you encourage your child to take the stairs instead of the elevator?” do not apply to inland Chinese cities.

In recent years, the topic of PAPP has attracted the attention of experts and parents in mainland China. Still, there is a general lack of theoretical foundations and qualitative formative research. To our knowledge, there is no widely accepted questionnaire for assessing PAPP in mainland China. In mainland China, the amount of time parents spend with their children is significantly reduced after children start school due to academic pressure or the nature of the school (e.g., boarding school). Therefore, this study aimed to develop and validate a questionnaire to assess the PAPP of parents of children aged 3–6 in mainland China.

## Methods

### Stage I: primary instrument development

#### Step one: determining the scale structure

A literature review was conducted to select three domains of general parenting theory as the basis for the scale structure. Dimensions and items from questionnaires or scales borrowed from related studies were identified. Subsequently, 12 parents were selected for semi-structured interviews. When choosing the sample, we considered maximum variability in gender, age, ethnicity, education level, marital status, and total monthly household income. After the interviewer explained the purpose of this study and obtained informed consent from the interviewee, the interview was started and recorded. The outline was as follows: (1) Have you been the primary caregiver for your child for the past year? (2) Do you understand the role of physical activity in children’s development? (3) Do you think there are ways to promote physical activity in your child? (4) What specific methods do you use to get your child moving? (5) Is there any relevant event that impresses you? After analyzing the data using Colaizzi’s 7-step analysis method, three verbal strategies were extracted: criticism, mockery, and gossip, which we categorized into the persuasion dimension. This step established a Chinese item pool with 10 dimensions (Table 1) and 123 candidate items.

**Table 1:** Initial conceptual framework and operational definitions.

Domain	Initial dimension	Number of items	Definition	Source of items
Control	Demands	11	Parents motivate children to participate in PA by applying pressure or prescribing appropriate punishments.	Larios et al. (2009) Mâsse et al. (2020) Timperio et al. (2008) O'Connor et al. (2014)
	Restriction	11	Parents discipline behaviors detrimental to their children’s PA by establishing rules.	Davison et al. (2011) Larios et al. (2009) Timperio et al. (2008) Vaughn et al. (2013)
	Permissive	6	Behavior in which the parent lacks demand (allowing the child to decide) on the children’s PA.	Mâsse et al. (2020)
Responsiveness	Education	10	Parents use strategies or behaviors that directly or indirectly (training) promote their children’s knowledge, understanding, and skills for PA.	Mâsse et al. (2020) O'Connor et al. (2014) McMinn et al. (2009) Vaughn et al. (2013)
	Empowerment	6	Parents improve their children’s physical activity or increase their opportunities for physical activity by providing human, material, financial, and transportation resources.	Davison et al. (2011) Mâsse et al. (2020) Vaughn et al. (2013)
	Persuasion	19	Parents use verbal strategies to elicit positive or negative feelings in their children, promoting action or reducing sedentary behavior.	Anderson and Coleman (2008) Brustad (1996) O'Connor et al. (2014) Vaughn et al. (2013) Parents interviews
	Autonomy promotion	16	Parents promote their children’s independent decision-making regarding PA by providing choices for or consulting with their children.	Mâsse et al. (2020) Suen et al. (2017)
	Motivation	12	Parents motivate their children to be rewarded and self-improvement by prescribing rewards and expressing expectations (related to physical activity).	Gubbels et al. (2011) Vaughn et al. (2013) Timperio et al. (2008)
Structure	Environmental support	13	Parents change residential and social environments to influence their children’s PA behaviors.	Vaughn et al. (2013) Suen et al. (2017) Parents interviews
	Modeling	19	Parents demonstrate positive behaviors related to PA for their children.	Larios et al. (2009) McMinn et al. (2009) Suen et al. (2017) Davison et al. (2011)

#### Step two: Delphi surveys

We organized two rounds of Delphi surveys and invited 15 experts (5 pediatric nurses, 5 pediatricians, 1 sports medicine physician, 2 educators, and 2 statisticians) to evaluate the item pool. The experts’ mean working experience was 25.08 ± 9.23 years, and their collective experience and knowledge ensured the robustness and relevance of the scale items. The experts were asked to evaluate the importance of the dimensions and items using a 5-point Likert scale ranging from 1 (very unimportant) to 5 (very important) and to self-assess their familiarity with the indicators and the basis for their judgments. Dimensions and items with mean scores >3.5 and coefficients of variation (CV) < 0.25 were retained. The experts were also asked to assess the accuracy and clarity of each dimension and item and to suggest specific deletions or modifications. We paid consulting fees to all of the experts.

The authority coefficient was 0.896, and all experts proposed amendments in the two rounds of correspondence. In the first round of correspondence, Kendall’s W values for the dimensions and items were 0.609 (*p <* 0.05) and 0.149 (*p* < 0.05), respectively. The experts identified “permissive” as the behavior opposite of “demands” and suggested deleting it. Furthermore, they identified the meanings of “empowerment” and “education” as overlapping and recommended merging these dimensions. In the second round of correspondence, the Kendall’s W values for the dimensions and items were 0.666 (*p* < 0.05) and 0.156 (*p* < 0.05), respectively. After the Delphi surveys, 52 items were deleted, 14 items were merged, 19 items were modified, and 3 new items were added, resulting in an item pool of 8 dimensions and 60 items.

#### Step three: pilot testing

To further assess the scale items’ clarity, comprehensibility, and feasibility, we conducted a pretest with 25 parents in the child healthcare department of a hospital in Xi’an using convenience sampling. The inclusion criteria were as follows: (1) the child’s father or mother, (2) the child’s primary caregiver (the leading participant who is responsible for the child’s daily living, such as eating, sleeping, and activities), (3) currently having at least one child aged 3–6 years, (4) the ability to read and understand Chinese, and (5) volunteering to participate in this study. The exclusion criteria were (1) children aged 3–6 years who had a disease or disability that limited participation in PA or (2) parents who had a disease or disability that limited participation in PA.

We recorded items that the respondents had questions about during the field survey. Each item on the scale measured the frequency of behaviors or strategies for parents in the past month. The response format for each item included five levels: 1 = never, 2 = seldom, 3 = sometimes, 4 = often, and 5 = always. The mean score was calculated as the sum of the items divided by the number of items answered in each dimension to calculate the PAPP scores. Twenty-five parents completed the questionnaire in 8–15 min. Based on participant feedback, the 2-item formulation was fine-tuned, and the informed consent form was refined, resulting in a CPAPPS I version that contained 8 dimensions and 60 items.

### Stage II: final instrument development

A cross-sectional research design was used for the item analysis and the reliability and validity tests. Five trained investigators collected the data for all three samples reported in this study from August 2022 to May 2023. The inclusion and exclusion criteria were the same as those for the pilot test. There was no data crossover between any of the three samples. According to the Kendall criterion, the number of respondents is roughly 5–10 times the number of scale items (Tang et al., 2023). The number of scale items used in the three surveys was 60, 40, and 30 in that order, and the minimum sample size should be 315, 210, and 175 cases, respectively, considering the 5% inefficiency rate.

#### Step one: item analysis

Item analysis determines whether each item should be eliminated or retained quantitatively. In this study, an item was removed if it met one or more of the following criteria: (1) the selection rate of an option for an item was >80%; (2) the critical ratio (CR) of the item was found to be insignificant; (3) the CV of the item was >15%; (4) the item total correlation coefficient was not significant, or the coefficient was <0.3; (5) the factor loading value of an item within each factor was <0.4 or the number of items contained in a factor was <3. The dimensions and items were deleted depending on the situation (Cao et al., 2019).

Sample 1 comprised 316 parents sampled randomly from three urban and two suburban kindergartens in Xi’an with the same inclusion and exclusion criteria used for pilot testing. The parents completed the CPAPPS-I under the guidance of the investigators. The response rate was 92%. After the analysis excluded the ceiling and floor data, 284 questionnaires were included (effective rate 90%). The mean age of the subjects was 35.94 ± 4.07 years. This sample was used to screen items for the CPAPPS-I. Twenty items that did not meet the above criteria were deleted. During factor analysis, the remaining items in the persuasion dimension were categorized into the education dimension. It is reasonable because persuasion is usually considered an educational tool in China. After this screening round, the CPAPPS-II comprised 40 items in 7 dimensions.

Sample 2 consisted of 412 parents randomly selected from 5 kindergartens in 3 cities (Xi’an, Yulin, and Yan’an) in Shaanxi Province. Online and on-site surveys were used for data collection. The response rate was 94%. After data cleaning, 388 questionnaires were included in the analysis (effective rate 94%). The mean age of the subjects was 35.85 ± 5.89 years. This sample was used for item screening of the CPAPPS-II.10 items removed in this screening round. Six common factors were extracted after factor analysis. The education, autonomy promotion, demands, and modeling dimensions were retained. The restriction of all items with factor loadings that were too low was removed. It may be that the preset items involved multiple independent factors, such as social rules, risk perception, and environmental adaptation, rather than a single “limiting behavior.” Items in the environmental support dimension (e.g., supervision, planned activities) may belong to different behavior types, resulting in a loose factor structure. Items related to planned activities (e.g., I will schedule family activities related to physical activity) were categorized in the role modeling dimension. Items related to safety (e.g., before my child is physically active, I will check with him/her to ensure that the environment or site is safe) were categorized into autonomy promotion. The motivation dimension was split into the reward and expectation dimensions. This process resulted in a formalized version of the CPAPPS with 30 entries in 6 dimensions.

#### Step two: preliminary validation of the final scale

Sample 3 included 945 parents of young children in 10 kindergartens in Xi’an, Shaanxi Province, Harbin, Heilongjiang Province, and Nanning, Guangxi Province, which are located in the central, northern, and southern regions of China, respectively. The survey was conducted using the final version of the CPAPPS, and the same data collection method was used for Sample 2. A total of 933 questionnaires were returned (response rate 98%), and 899 were included in the analysis (effective rate 93%). This sample was used to explore the structure and to evaluate the validity and reliability of the final CPAPPS. Fifty parents out of the 899 selected subjects randomly repeated the questionnaire after 1 month. There were no differences in the distribution of gender or age between those who completed the second questionnaire and those who did not.

The data were analyzed using SPSS 26.0 and AMOS 25.0 in this step. Descriptive statistics were used for demographic variables and overall and individual scores. A t-test or one-way ANOVA was used to compare the CPAPPS scores of different groups, and a difference of *p* < 0.05 was considered statistically significant.

### Validity analysis

#### Construct validity

The data from 899 cases were divided into two groups based on the principle of odd and even columns. Of these, 450 cases were subjected to EFA to extract the factor structure using principal component analysis and maximum variance rotation. The other 449 samples were subjected to validation factor analysis (CFA) to verify whether the preset theoretical factor model was suitable for the actual data. Before EFA was conducted, the Kaiser–Meyer–Olkin (KMO) test and Bartlett’s sphere test were used to measure the suitability of the sample for factor analysis. The data were suitable for EFA because the KMO value was >0.6, and Bartlett’s ball-point test result was *p* < 0.05.

The eigenvalues and the scree plot determined the number of factors. The maximum factor that should be extracted is the first point at which Cattell’s scree plot begins to flatten. Items were retained if they met the following criteria: (1) factor loadings >0.4 and no cross-factors, and (2) items were conceptually consistent with their corresponding factors. CFA was subsequently performed. Since some observed variables were non-normally distributed, the general least squares (GLS) method was used to estimate the model parameters.

The CMIN/DF, goodness of fit index (GFI), adjusted goodness of fit index (AGFI), Tucker–Lewis index (TLI), normed fit index (NFI), and root mean square error of approximation (RMSEA) were calculated to assess how well the model fits the data, with CMIN/DF < 3, GFI, AGFI, TLI, NFI > 0.90, and RMSEA <0.08 indicating good model fit. In addition, the average variance extracted (AVE), construct reliability (CR), and correlation coefficients between factors were calculated to validate the discriminant and convergent validity of the subfactors of the tool. AVE > 0.7 and CR > 0.5 indicated good convergent validity, and the square root of the AVE was larger than the correlation coefficient between factors, indicating good discriminant validity (Fazhan et al., 2023).

#### Concurrent validity

Research (Zhou et al., 2023) shows significant differences in parenting behaviors with different parenting motives, and early parenting behaviors that parents with misaligned parenting motives adopt are inappropriate. Parenting motivation and parenting behavior are correlated; parenting motivation is an upstream variable of parenting behavior. Because PA parenting is an aspect of parenting behavior, this study used PA parenting motivation as an index to evaluate validity. Chinese scholars developed the Self-Regulation Questionnaire (SRQ), to measure the degree of internalization of parenting motivation, which was used to assess validity (CMIN/DF = 3.89, CFI = 0.90, IFI = 0.90, GFI = 0.88, NFI = 0.88, RMSEA = 0.07, Cronbach’s *α* = 0.90) (Zhou et al., 2023). The degree of internalization of parenting motivation was measured by the relative autonomy index (RAI), with higher RAI scores indicating more autonomous parenting motivation and lower RAI scores indicating more controlled parenting motivation. Concurrent validity was assessed by Spearman correlation coefficients between the two scale scores and factor scores.

#### Reliability analysis

Reliability was assessed by Cronbach’s alpha coefficient, the split-half reliability coefficient, and the test–retest reliability coefficient. Reliability coefficients >0.70 were considered satisfactory (Cao et al., 2019).

### Data collection and quality control

To conduct the survey, we first contacted the director of the kindergarten. After we obtained the director’s consent, the director informed the classroom teachers of each classroom and asked them to cooperate with the researcher in conducting the survey. The data for Sample 1, Sample 2, and Sample 3 in the Xi’an area were collected via field surveys. The parents of the children selected from each class were organized in one classroom. After the investigator explained the purpose and requirements of the study in detail, the parents completed the questionnaire, which the investigator subsequently collected. Data for Sample 2 and Sample 3 were collected outside of Xi’an using an electronic questionnaire, and quality control was achieved through the following steps. (1) The first page was an informed consent page that explained the content and purpose of the study. The scale could be completed after this page was read. (2) A hint was presented for missing answers, and the survey could not be submitted without them. (3) Important content was highlighted in bold and in eye-catching colors. (4) Large font was used to visually present the scoring options to make them easier to complete. (5) The classroom teacher distributed Detailed explanations and unified guidelines to the parents who completed the survey. (6) The app screened and cleaned the data, checked the answers, and eliminated random answers or answers with a response time of less than 4 min to ensure the accuracy and completeness of the information entered.

Two people entered the data collected from the field survey into a database using EpiData3.1. The data were checked for logic, and incomplete questionnaires were eliminated.

## Results

### Demographic characteristics of the sample

The demographic characteristics of Samples 1, 2, and 3 are shown in Table 2. Differences in demographic characteristics such as gender, age, education, ethnicity, marital status, place of residence, and monthly household income were not statistically significant for parents in the EFA and CFA samples.

**Table 2:** Demographic characteristics of participants.

Characteristics	Sample1 (*n* = 284)	Sample2 (*n* = 388)	Sample3 (*n* = 899)	EFA (*n* = 450)	CFA (*n* = 449)	*χ*^2^/*Z*	*P*
Gender							
Male (father)	76	83	347	173	174	0.009	0.924
Female (mother)	208	305	552	277	275		
Age (M ± SD)	35.94 ± 4.07	35.85 ± 5.89	35.09 ± 6.06	34.83 ± 5.93	35.37 ± 6.19	−1.310	0.191
Ethnic groups						1.343	0.246
Han people	272	369	771	392	379		
Minority people	12	19	128	58	70		
Marital status						1.488	0.475
Single	3	18	35	20	15		
Married	281	370	864	430	434		
Education						−0.135	0.892
Junior high school and below	1	62	178	93	85		
High School/Junior College	15	59	162	77	85		
College	27	70	217	111	106		
Undergraduate	158	154	296	142	154		
Master and above	83	43	46	27	19		
Residence						−1.245	0.210
City	262	274	576	279	297		
Suburbs/Town	22	43	212	89	83		
Rural	0	71	111	65	46		
Monthly household income						−1.277	0.202
<5,000	16	133	405	213	192		
5,000 ~ 10,000	52	102	200	96	104		
10,000 ~ 20,000	122	94	163	73	90		
>20,000	94	59	131	68	63		
Family structure						−1.686	0.093
Nuclear family	134	205	635	307	328		
Main family	145	162	175	92	83		
Single parent family	5	21	89	45	44		

### Assessing the psychometric properties of the scale

#### Construct validity by EFA

The EFA was performed on the data of 450 cases from Sample 3. The Kaiser–Meyer–Olkin coefficient of sampling adequacy was 0.863, the approximate chi-square value of Bartlett’s test of sphericity was 8405.11, and the probability was lower than 0.05. The results indicated that the sample size was sufficient and suitable for factor analysis.

**Figure 1:**
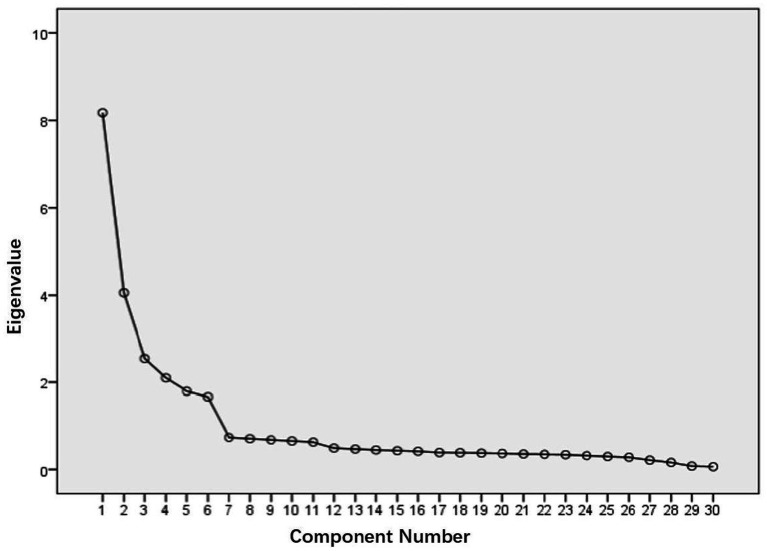
Scree plot of principal component factor analysis (*n* = 450).

The scale was explored by extracting common factors with an eigenvalue more significant than one and limiting the number of factors to 6. The results revealed that the overall structure was favorable with a factor of 6, which was corroborated by the scree plot (Figure 1). These 6 factors explained 67.962% of the total variance. All items were maintained in the original dimensions of the formal version of the CPAPPS, and the factor loadings for all items were higher than 0.672 with no cross-loadings (Table 3).

**Table 3:** Results of the exploratory factorial analysis.

Dimension name and items	Factor loading	Communality
Factor 1: education (eigenvalue = 7.964, % of variance = 26.584%)		
A1 I teach my child new or different programs or games that will get him/her active	0.950	0.945
A2 I instruct children in PA (e.g., how to jump rope)	0.793	0.652
A3 I answer my child’s questions about PA.	0.808	0.688
A4 I tell my child that he/she can make new friends through PA.	0.811	0.692
A5 I tell my child to choose PAs appropriate for his/her developmental level.	0.794	0.659
A6 I try to make PA more fun for my child so that he/she enjoys it more.	0.798	0.657
A7 I set goals to encourage my child to be more physically active.	0.805	0.687
A8 I encourage my child to play more active games.	0.844	0.743
Factor 2: autonomy promotion (eigenvalue = 4.16, % of variance = 13.865%)		
F1 I ask the child to name or list his/her favorite PA himself/herself	0.946	0.928
F2 I ask my child what PA he/she would like to do before the activity.	0.786	0.636
F3 I chose PA, which I can participate in with my child.	0.776	0.631
F4 In my free time, I let my child decide when to start the PA.	0.805	0.662
F5 When the whole family is physically active together, I will let my child choose exactly what to do	0.794	0.653
F6 I let my child choose the location or place for PA.	0.774	0.642
F7 I check with my child that the environment or site is safe before PAs.	0.81	0.671
F8 I regularly check and maintain my child’s sports equipment or toys with him/her.	0.772	0.628
Factor 3: demands (eigenvalue = 2.659, % of variance = 8.864%)		
D1 I punish my child appropriately if he/she refuses to do PA.	0.891	0.843
D2 I will ask my child to go outdoors because I feel that only outside can my child be active.	0.727	0.538
D3 I enroll my child in a sports class because I feel that only this organized training can make my child active.	0.72	0.556
D4 I ask my child to be physically active to improve his/her physical fitness.	0.674	0.484
D5 I ask my child to train to be good at a particular sport.	0.672	0.489
Factor4: modeling (eigenvalue = 2.103, % of variance = 7.01%)		
E1 I am in the habit of regular PA.	0.908	0.888
E2 I show my child that I am physically active.	0.847	0.74
E3 I talk to my child about how much I enjoy being physically active	0.828	0.725
Factor5: expectations (eigenvalue = 1.762, % of variance = 5.873%)		
C1 I want my child to play outside if the weather is good.	0.845	0.788
C2 I want my child to be good at a particular PA.	0.771	0.607
C3 I hope my child to be physically active for 1 h or more every day.	0.789	0.64
Factor 6: rewards (eigenvalue = 1.741, % of variance = 5.802%)		
B1 I reward my child for being physically active (e.g., initiating PA, initiating participation in sports).	0.85	0.773
B2 I reward my child for progress/achievement in physical activity.	0.752	0.572
B3 I use outdoor activities to reward good behavior (e.g., telling my child I will take you to the park if you learn to brush your shoes).	0.729	0.573

#### Construct validity by CFA

**Figure 2:**
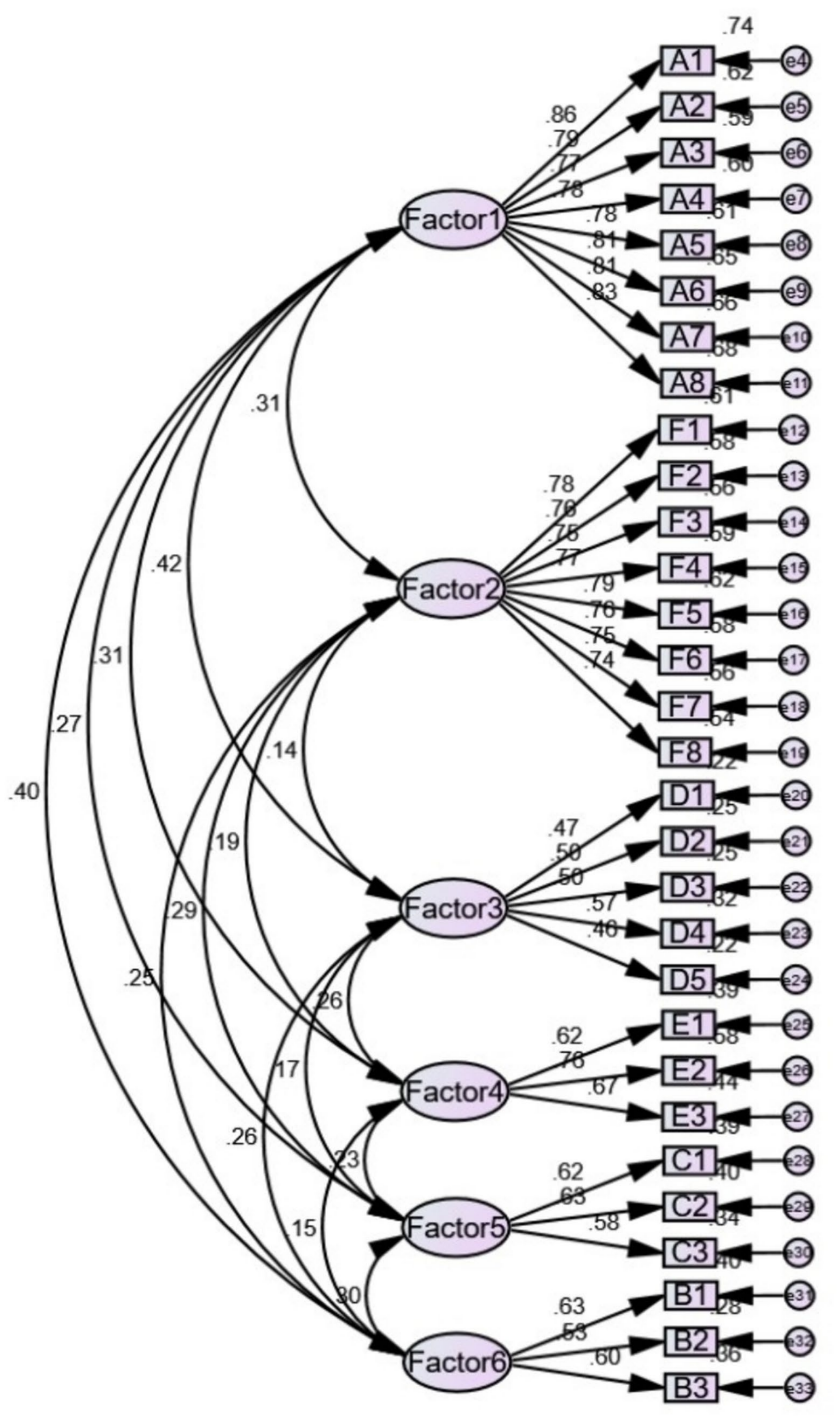
The standardized path diagram of the confirmatory factor analysis. A1 to F8 represent the items of CPAPPS, and Factor 1 to Factor 6 are the 6 factors of CPAPPS.

The central path diagram of the model was plotted based on the EFA results, which revealed that CMIN/DF = 1.147, GFI = 0.938, AGFI = 0.927, TLI = 0.989, NFI = 0.926, and RMSEA = 0.018, indicating a satisfactory model fit. The standardized factor loadings for each item were >0.40, which was statistically significant. The parameter estimates for the CFA are shown in Figure 2. The AVE for the six factors was >0.5 (0.645, 0.747, 0.774, 0.758, 0.769, and 0.791, respectively). The CR of each factor was >0.7 (0.936, 0.713, 0.741, 0.627, 0.724, and 0.920, respectively). The square root of the AVE was more significant than the correlation coefficients among the 6 factors, as shown in Table 4, indicating that the CPAPPS had good convergent and discriminant validity.

**Table 4:** Results of the confirmatory factorial analyses.

Factor	Education	Autonomy promotion	Demands	Modeling	Expectations	Rewards
Education (Factor 1)	0.803*					
Autonomy promotion (Factor 2)	0.298	0.589*				
Demands (Factor 3)	0.207	0.184	0.611*			
Modeling (Factor 4)	0.316	0.165	0.109	0.508*		
Expectations (Factor 5)	0.259	0.114	0.165	0.188	0.685*	
Rewards (Factor 6)	0.286	0.188	0.224	0.112	0.161	0.769*

* represents the square root of AVE of 6 factors; the others represent the correlation coefficients between 6 factors.

Overall, the results showed an acceptable model fit in a test sample of Chinese parents of children aged 3–6 years. The results of the EFA and CFA supported the structural validity of the CPAPPS.

#### Concurrent validity

The CPAPPS and SRQ scores were non-normally distributed. Spearman’s rank correlation analysis revealed a moderate positive correlation between the total CPAPPS and SRQ scores (*r* = 0.541, *p* < 0.05). There was a positive correlation between each CPAPPS dimension and the total score of the SRQ, with correlation coefficients ranging from 0.206 to 0.270; these were all low-degree correlations. This result reflects the independence of the scale. The highest correlation coefficient was found between parental autonomy motivation and modeling in the CPAPPS (*r* = 0.322, *p* < 0.05), indicating that the more autonomous a parent is, the greater the correlation between parenting motivation and parenting practices in terms of teaching by example. Except for a lack of correlation with the education dimension, parents’ controlling motivation was negatively correlated at a low degree with all other dimensions of the CPAPPS, with correlation coefficients ranging from −0.167 to 0.060 (*p* < 0.05). Overall, the validity of the CPAPPS was acceptable, as shown in Table 5.

**Table 5:** The correlation between the scores of CPAPPS and SRQ.

SQR	CPAPPS						
	Education	Rewards	Expectations	Demands	Modeling	Autonomy promotion	Total score
Autonomy motivation	0.319**	0.261**	0.278**	0.297**	0.322**	0.312**	0.662**
Controlling motivation	−0.060	−0.106**	−0.137**	−0.137**	−0.167**	−0.139**	−0.307**
Total score	0.222**	0.206**	0.231**	0.244**	0.270**	0.251**	0.541**

** represents *p* < 0.01.

#### Reliability analysis

The Cronbach’s alpha coefficient for the CPAPPS was 0.892, and the Cronbach’s alpha coefficients for the dimensions ranged from 0.714 to 0.942. The split-half reliability coefficient for the CPAPPS was 0.658, and the dimensions ranged from 0.671 to 0.945. The retest reliability coefficient for the 50-subject subsample at four-week intervals was 0.844, ranging from 0.695 to 0.891 for all dimensions (Table 6).

**Table 6:** Reliability of the CPAPPS.

Dimensions	*N*	Cronbach’s *α*	Guttman split-half reliability	Test–retest reliability
Education	8	0.942	0.945	0.863
Rewards	3	0.714	0.671	0.695
Expectations	3	0.758	0.738	0.891
Demands	5	0.806	0.787	0.827
Modeling	3	0.858	0.827	0.797
Autonomy promotion	8	0.932	0.927	0.784
Total	30	0.892	0.658	0.844

#### Discriminant ability

Table 7 shows the CPAPPS scores of parents by gender, age, ethnicity, marital status, education, residence, and monthly family income. There was no difference between fathers’ and mothers’ scores on each dimension (*p* < 0.05). The demand dimension scores differed significantly (*p* < 0.05) between parents younger than 30 years old and parents 40–50 years old. Differences in parenting practices regarding rewards and expectations were statistically significant among parents of different ethnicities (*p* < 0.05). Compared with married parents, parents who were currently single (divorced, widowed, or unmarried) had lower scores for education, rewards, modeling, and autonomy support (*p* < 0.05). There were significant differences in parents’ scores across different places of residence in all dimensions (*p* < 0.05). There were no significant differences in the scores of parents with different monthly household incomes and family structures on each dimension (*p* < 0.05).

**Table 7:** Comparison of CPAPPS scores among different groups (*n* = 899).

Group	Education	Rewards	Expectation	Demands	Modeling	Autonomy promotion
Gender						
Male (father)	2.48 ± 0.84	2.88 ± 0.75	2.92 ± 0.83	2.88 ± 0.73	2.79 ± 0.88	2.65 ± 0.79
Female (mother)	2.38 ± 0.85	2.93 ± 0.81	2.88 ± 0.83	2.83 ± 0.70	2.78 ± 0.89	2.68 ± 0.76
Age (years)						
<30	2.45 ± 0.84	2.90 ± 0.77	2.95 ± 0.84	2.91 ± 0.72	2.77 ± 0.88	2.70 ± 0.79
30–40	2.42 ± 0.85	2.92 ± 0.79	2.86 ± 0.81	2.85 ± 0.71	2.82 ± 0.87	2.65 ± 0.78
40–50	2.37 ± 0.85	2.89 ± 0.80	2.89 ± 0.87	2.76 ± 2.69^a^	2.73 ± 0.92	2.67 ± 0.73
Ethnic groups						
Han people	2.43 ± 0.85	2.93 ± 0.78	2.87 ± 0.83	2.85 ± 0.71	2.78 ± 0.88	2.66 ± 0.78
Minority people	2.37 ± 0.82	2.77 ± 0.81^b^	3.05 ± 0.84^b^	2.82 ± 0.73	2.85 ± 0.93	2.71 ± 0.71
Marital status						
Single	1.98 ± 0.56	2.46 ± 0.93	2.66 ± 0.84	2.66 ± 0.72	2.21 ± 0.90	2.42 ± 0.70
Married	2.43 ± 0.85^c^	2.93 ± 0.77^c^	2.90 ± 0.83	2.86 ± 0.71	2.81 ± 0.88^c^	2.68 ± 0.77^c^
Education						
Junior high school and below	2.36 ± 0.84	2.90 ± 0.79	2.96 ± 0.83	2.80 ± 0.72	2.74 ± 0.93	2.69 ± 0.78
High School/Junior College	2.42 ± 0.86	2.85 ± 0.84	2.87 ± 0.78	2.82 ± 0.72	2.69 ± 0.89	2.60 ± 0.77
College	2.36 ± 0.84	2.96 ± 0.76	2.89 ± 0.83	2.93 ± 0.66	2.80 ± 0.93	2.62 ± 0.78
Undergraduate	2.48 ± 0.85	2.90 ± 0.78	2.83 ± 0.87	2.84 ± 0.73	2.82 ± 0.81	2.71 ± 0.77
Master and above	2.46 ± 0.84	2.91 ± 0.79	3.13 ± 0.03^d^	2.88 ± 0.71	3.01 ± 0.89^e^	2.74 ± 0.77
Residence						
City	2.46 ± 0.85	2.94 ± 0.78	2.93 ± 0.84	2.90 ± 0.71	2.83 ± 0.90	2.70 ± 0.78
Suburbs/Town	2.40 ± 0.79	2.82 ± 0.82	2.87 ± 0.69	2.78 ± 0.70	2.74 ± 0.91	2.69 ± 0.73
Rural	2.29 ± 0.84^f^	2.86 ± 0.77^f^	2.82 ± 0.86^f^	2.75 ± 0.71^f^	2.68 ± 0.83^f^	2.56 ± 0.77^f^
Monthly income						
<5,000	2.42 ± 0.87	2.93 ± 0.81	2.92 ± 0.83	2.81 ± 0.73	2.83 ± 0.90	2.69 ± 0.77
5,000 ~ 10,000	2.44 ± 0.81	2.90 ± 0.74	2.82 ± 0.85	2.91 ± 0.72	2.76 ± 0.89	2.61 ± 0.81
10,000 ~ 20,000	2.42 ± 0.85	2.81 ± 0.81	2.91 ± 0.81	2.90 ± 0.64	2.77 ± 0.87	2.70 ± 0.75
>20,000	2.35 ± 0.82	2.96 ± 0.73	2.91 ± 0.84	2.80 ± 0.69	2.71 ± 0.87	2.64 ± 0.72
Family structure						
Nuclear family	2.42 ± 0.85	2.91 ± 0.77	2.91 ± 0.84	2.85 ± 0.71	2.79 ± 0.89	2.66 ± 0.78
Main family	2.38 ± 0.90	2.94 ± 0.86	2.87 ± 0.83	2.88 ± 0.69	2.83 ± 0.88	2.72 ± 0.75
Single parent family	2.48 ± 0.65	2.83 ± 0.72	2.82 ± 0.83	2.75 ± 0.77	2.63 ± 0.87	2.59 ± 0.73

a represents *p* < 0.05 vs. age lower than 30 years, b represents *p* < 0.05 vs. Han people, c represents *p* < 0.05 vs. Single, d represents *p* < 0.05 vs. Undergraduate, e represents *p* < 0.05 vs. High School/Junior College, f represents *p* < 0.05 vs. City.

## Discussion

In this study, we developed the Chinese Physical Activity Parenting Practices Scale using general parenting theories as a framework. The scale contains 30 items in 6 dimensions: education, autonomy support, rewards, expectations, modeling, and demands. The scale is reliable and valid and can be used to study Chinese parents’ physical activity parenting practices of children aged 3–6.

In China, the government has attached great importance to the physical fitness of children and adolescents. In recent years, several policies have been introduced to promote improvements in children’s and adolescents’ PA levels. However, distal externalities have had little success in counteracting the dramatic effects of lifestyle changes, and children’s and adolescents’ PA levels continue to decline. Research confirms that children have a natural intrinsic interest in PA, and perceptions and habits of PA established in childhood tend to carry over into adulthood and impact adult health (Zeng et al., 2023). Meanwhile, the dynamic interaction that exists between physical activity parenting practices and children’s physical activity levels has been confirmed by a variety of theories. Social cognitive theory emphasizes that parents shape children’s activity patterns through behavioral modeling and reinforcement mechanisms. A study using accelerometers showed that children’s moderate-vigorous activity (MVPA) attainment increased by 58% when parents engaged in family exercise ≥3 times per week (Trost et al., 2013). On the other hand, ecological systems theory reveals synergies between family microsystems and extra-community systems. Studies have shown that structured parenting practices (e.g., shared exercise planning) increase children’s MVPA attainment by 2.3-fold in safe community settings (Sleddens et al., 2012). Notably, self-determination theory proposes that autonomy-supportive parenting effectively promotes children’s intrinsic motivation to exercise, leading to significant increases in exercise (Ntoumanis et al., 2021). Therefore, it is essential to rely on parents as the key socialization agents to plant the “seeds of love for PA” early in parenting and to transform children’s intrinsic interest in PA into an intrinsic motivation to engage in PA through specific parenting practices. Children between the ages of 3 and 6 have already acquired a certain level of physical mobility and understanding. They spend most of the day with their parents and maintain a close and dependent relationship. Therefore, Parents have a more significant influence on children at this stage.

The final structure of the CPAPPS was based on the CFA validation results. In contrast to the activity support scale for multiple groups (ASSMG) (Davison et al., 2011), our scale did not extract the logistical support dimension. This may be because our sample population came from different regions (e.g., urban, township, rural). Logistical support included items that reflect the family’s financial situation, such as enrolling a child in basketball classes, which requires parents to pay the appropriate fees. Some parents from townships and rural areas find it difficult to afford this cost. In addition, rural education resources are limited, and parents may face a lack of access even if they are willing to spend money. We believe that emphasis should be placed on PA for parents and children from weaker economic backgrounds to develop recommendations for PA parenting practices that apply to a broad range of people. The restriction dimension was removed from the initial conceptual framework, which is inconsistent with the PAPP, APQ, and PARR scales. Most items in this dimension were safety-motivated restrictions, such as supervising children when playing outdoors. For children aged 3 to 6 years, Chinese parents believe that safety is a prerequisite for all activities. Moreover, since most residents of cities and townships in China live in apartment buildings without separate yards, children playing outdoors are inevitably accompanied by adults.

After we conducted CFA, the dimensions of education, empowerment, and persuasion were merged into a single structure because empowerment and persuasion are different ways of education. The motivation dimension was divided into rewards and expectations, which is consistent with the PAPP item bank. Conceptually, this split regrouped items that focused on material rewards. In contrast, although expectations are considered the most long-term approach to motivation (Shang et al., 2023), they focus on the psychological dimension and are a more substantial attribute of parenting strategies. These similarities and differences reflect the differences and commonalities of PA parenting practices in different countries and regions.

Differences in family roles assumed by people of different genders result in differences in the performance of parenting practices. Multiple studies have shown that fathers significantly influence role modeling more than mothers (Neshteruk et al., 2017; Sigmund et al., 2018). A survey of Irish parents revealed that mothers and fathers differed in their roles concerning PA and that fathers were more involved in parenting practices (Sohun et al., 2021). Our study did not observe a significant difference between fathers and mothers regarding parenting practices across PA. The difference in the demand dimension was significant (*p* < 0.05) between parents younger than 30 years old and parents aged 40–50 years old. This phenomenon may be because older parents are more emotionally stable, have a compensatory mentality for having children later in life, or have energy constraints that lead to a greater preference for non-controlling parenting practices (Fang et al., 2024). Such low requirements may weaken children’s sense of rules and resistance to frustration, which is not conducive to establishing good physical activity habits.

In the comparison of parenting practices among different ethnic groups, although there were significant differences only in the dimensions of rewards and expectations, we also observed that Han parents scored higher than ethnic minority parents in the dimensions of education, rewards, and demands and lower than ethnic minority parents in the dimensions of expectations, modeling, and autonomy support. These results reflect the cultural differences between Han people and ethnic minorities. As ethnic minorities live in areas with more variable natural environments, such as mountainous regions and plateaus, their lifestyles and cultural traditions make them more active in their daily lives. Han culture is based on Confucianism, emphasizing ritual norms and ideological aspects. We also found that in our sample population, single fathers or mothers used parenting practices that focused on education, rewards, modeling, and autonomy support less frequently than married parents did. This finding is consistent with a recent study of parents in Hong Kong, China. It may be related to the support parents receive from their partners and the additional time and energy to devote to parenting activities (Suen et al., 2019).

Examining the relationship between parenting practices and children’s motor behaviors requires considering the physical and social environments in which families live (Goncalves et al., 2020). Our study revealed that parents who lived in urban areas used all parenting practices significantly more often than those in rural areas. As mentioned earlier, parents from rural areas were not comparable to those who lived in urban areas regarding awareness, time, income, activity venues, and the accessibility of organized training. This finding also underscores the urgent need to develop effective parenting interventions for rural parents to enhance their PA-related parenting skills. These results support the good discriminant validity of our scale.

Although the dimensions were reduced from 10 to 6 compared with the initial conceptual framework, the scale still covers the three domains of general parenting theories and standard PA-related parenting practices of Chinese parents of 3- to 6-year-old children. In practical applications, CPAPPS can be used to design multi-tiered strategies in conjunction with scenario characteristics. In school programs, CPAPPS can be used as a baseline assessment tool to identify parents’ parenting practices in encouraging physical activity and setting an example. In turn, personalized feedback is provided through parent workshops to guide the development of a home exercise program synergistic with school sports activities. In public health campaigns, CPAPPS data can help locate weaknesses in community parenting practices. For example, family exercise kits can be distributed in areas where “structural environment creation” is inadequate, and media campaigns can be used to reshape social norms. For weight management of overweight/obese children, CPAPPS can effectively identify risk factors such as poor parenting practices. Clinical interventions can be designed with milestones based on the dimensions of the scale, such as cognitive restructuring to enhance parental autonomy, training in positive reinforcement skills, and integrating nutritional education to form a comprehensive program.

### Limitations

Despite the rigorous methodology used in this study to assess reliability and validity, several limitations exist. First, some data in sample 3 were collected online, requiring parents to have a smartphone to access the survey. Some parents with low income who did not have a cell phone or who could not operate a cell phone may have been excluded, which may have affected the generalizability of the data. Second, parent-reported parenting practices are at risk of social desirability bias and difficulty recalling problems associated with specific behaviors. The answers may, therefore, overestimate PA parenting practices that are perceived as positive or good. Third, we conducted testing only in three regions in China; thus, in further studies, researchers can extend the samples to other areas of China or countries. Fourth, we only used Classical Testing Theory to confirm the validity and reliability of the tool. In subsequent studies, we hope to validate the scale using other methods and tests.

## Conclusion

In this study, we present the Chinese Physical Activity Parenting Practices Scale. To our knowledge, this is the first questionnaire based on general parenting theory developed to assess the PA parenting strategies and behaviors of parents of 3- to 6-year-old children in mainland China. The questionnaire satisfies all psychometric properties and can be used as a practical tool to assess PA parenting practices in mainland China.

## Data Availability

The raw data supporting the conclusions of this article will be made available by the authors, without undue reservation.
